# Identifying dietary differences between Scotland and England: a rapid review of the literature

**DOI:** 10.1017/S1368980017001380

**Published:** 2017-07-20

**Authors:** Stephanie Chambers, Karen L Barton, Viviana Albani, Annie S Anderson, Wendy L Wrieden

**Affiliations:** 1 MRC/CSO Social and Public Health Sciences Unit, University of Glasgow, 200 Renfield Street, Glasgow G2 3QB, UK; 2 Division of Food and Drink, Abertay University, Dundee, UK; 3 Human Nutrition Research Centre and Institute of Health and Society, Newcastle University, Newcastle upon Tyne, UK; 4 Centre for Public Health Nutrition Research, University of Dundee, Dundee, UK

**Keywords:** Food consumption, Nutrient intake, Scotland, England, Mortality

## Abstract

**Objective:**

Rates of premature mortality have been higher in Scotland than in England since the 1970s. Given the known association of diet with chronic disease, the study objective was to identify and synthesise evidence on current and historical differences in food and nutrient intakes in Scotland and England.

**Design:**

A rapid review of the peer-reviewed and grey literature was carried out. After an initial scoping search, Medline, CINAHL, Embase and Web of Science were searched. Relevant grey literature was also included. Inclusion criteria were: any date; measures of dietary intake; representative populations; cross-sectional or observational cohort studies; and English-language publications. Study quality was assessed using the Quality Assessment Tool for Observational Cohort and Cross-sectional Studies. A narrative synthesis of extracted information was conducted.

**Results:**

Fifty publications and reports were included in the review. Results indicated that children and adults in Scotland had lower intakes of vegetables and vitamins compared with those living in England. Higher intakes of salt in Scotland were also identified. Data were limited by small Scottish samples, difficulty in finding England-level data, lack of statistical testing and adjustment for key confounders.

**Conclusions:**

Further investigation of adequately powered and analysed surveys is required to examine more fully dietary differences between Scotland and England. This would provide greater insight into potential causes of excess mortality in Scotland compared with England and suitable policy recommendations to address these inequalities.

Scotland is in the unenviable position of experiencing the highest age-standardised mortality rates and lowest life expectancy in Western Europe^(^ ^1^ ^)^. Although health outcomes have improved in the last 20 years, with premature mortality in those under 75 years of age dropping by over one-third, the gap between the overall age-standardised death rates from all causes for Scotland and the UK as a whole^(^ ^2^ ^)^ has not reduced. The gap remains at over 100 additional deaths per 100 000 individuals for Scotland, compared with the UK average.

Higher deprivation rates can explain some of these differences, but more than three-quarters of excess deaths cannot be accounted for through this explanation alone^(^ ^3^ ^)^. Further support for examining alternative explanations comes from research that compared the largest Scottish city, Glasgow, with two English cities (Liverpool and Manchester) with similar rates of deprivation and life expectancy. The results from that work highlighted that Glasgow experienced 30 % more premature deaths and 15 % more total deaths than these two cities^(^ ^4^ ^)^.

These large differences have received considerable critical attention in the literature, with a number of explanations put forward to explain the gap^(^ ^5^ ^)^, such as historically high levels of deprivation, regional economic policies, de-industrialisation and low levels of social capital^(^ ^6^ ^)^. The impact of diet and nutrition on health outcomes and life expectancy is of little doubt. Evidence identifies diet and obesity as key factors in CVD, diabetes^(^ ^7^ ^)^ and some of the most common cancers^(^ ^8^ ^)^. Nevertheless, dietary differences in relation to Scotland and England, and more specifically Glasgow and similar English cities, have not been investigated adequately. It is estimated that if the Scottish diet were similar to that consumed in England, then potentially 40 % of excess deaths could be avoided^(^ ^9^ ^)^. Comparing three years of food and nutrient data from the Family Food Survey, Scarborough *et al*.^(^ ^9^ ^)^ found that Scottish households were eating less fruit and vegetables, and more fat, saturated fat and salt, than English households.

## Policy context

At a policy level, the need to tackle poor diet and obesity in Scotland has been recognised; however, progress in dietary change is slow^(^ ^10^ ^)^. In addition, large inequalities exist in the nutritional quality of diets^(^ ^11^ ^)^, contributing to the risk of chronic diseases and obesity, the rates of which are higher in areas of deprivation^(^ ^12^ ^)^. The Scottish Dietary Targets^(^ ^13^ ^)^, reconfigured as Dietary Goals^(^ ^14^ ^)^, aim to increase fruit, vegetable, whole grains and fish intakes, and reduce saturated fat and added sugar; however, dietary change remains elusive^(^ ^10^ ^,^ ^11^ ^)^. Given the evidence that deprivation alone does not explain the higher mortality rates observed for Scotland, a key concern for the Scottish Government is to understand what other factors influence the higher prevalence of chronic disease as an important contributor to lower life expectancy and greater burden on local health services. Examining the historical and current dietary differences between Scotland and England in the published literature provides an opportunity to identify key areas for action and further examination.

## Aim

The present work was prepared in response to a commissioned call from NHS Health Scotland, a Scottish health board with a national remit to improve health and reduce inequalities. NHS Health Scotland commissioned a rapid review^(^ ^15^ ^)^ to identify and synthesise evidence on current and historical differences in food and nutrient intakes in Scotland and England (including differences between the cities of, and regions surrounding, Glasgow, Liverpool and Manchester). It is this work that the current paper reports.

## Methods

### Study design

Rapid review is an evidence synthesis methodology that applies a systematic approach to evidence identification and syntheses, but with a more limited scope than a systematic review. Rapid reviews generally seek a response to a policy or a clinically important query in a defined time period, working closely with the stakeholders seeking the answer to the query^(^ ^16^ ^)^. The need to draw together conclusions from the evidence in a timely manner impacts on the review’s precision^(^ ^17^ ^,^ ^18^ ^)^. Rapid reviews range in the methods used and the time period for completion, with some completed within 3 weeks and others taking as long as 6 months^(^ ^18^ ^)^. The present review was carried out over a 4-month period, and limited its scope by looking at four key databases, including English-language publications only, and by carrying out a restricted search of the grey literature.

### Search strategy

An initial scoping search was carried out. This involved identifying key dietary surveys from across the UK via the authors’ expertise and online searches. Google Scholar was also searched using ‘Scotland’, ‘England’, ‘Diet’, ‘Glasgow’, ‘Manchester’ and ‘Liverpool’ as search terms. The keywords of identified studies were then used to create the search terms for the main study searches. Searches were run in four databases from database start dates (Medline, 1946; CINAHL, 1937; Embase, 1947; Web of Science, 1945) until October 2014, using search terms specific to each database (see online supplementary material, Supplemental Tables 1–4). The wide date range was essential for examining historical dietary differences. Search terms were built around the location of the study sample, diet and nutrition outcomes, and study design, specifically population-based observational studies. Medical Subject Heading (MeSH) terms were used for Medline, and Subject Headings for CINAHL and Embase. Inclusion and exclusion criteria were defined to enable publication selection (Table 1).

**Table 1:** Inclusion and exclusion criteria for the present rapid literature review on dietary differences between Scotland and England

Inclusion	Exclusion
All years	Non-English language publications
Cross-sectional and observational cohort studies (including longitudinal studies such as birth cohort studies)	Randomised controlled trials, quasi-experimental studies, case–control studies, qualitative studies
Representative populations from the whole of England, the whole of Scotland, or the cities and surrounding areas of Glasgow, Liverpool or Manchester (including any studies representative of any age (adults or children) or gender strata)	Samples not designed to be representative of the overall population (except age/gender strata including children)
Includes measures of food intake or purchasing including:	Conference abstracts
1. Diet	
2. Energy intake	
3. Fruit and vegetable consumption	
4. Fat and saturated fat intakes	
5. Added sugar/NMES intakes	
6. Vitamin and mineral intakes	
7. Consumption of foods and drinks high in sugar	
8. Consumption of foods high in fat	
9. Consumption of foods high in salt	

NMES, non-milk extrinsic sugars.

Grey literature searching included searches of key websites and liaison with National Health Service and Local Authority contacts in Glasgow, Liverpool and Manchester. Websites included the sites of UK (e.g. National Diet and Nutrition Survey, Low Income Diet and Nutrition Survey), English (e.g. Health Survey for England) and Scottish (e.g. Scottish Health Survey) national surveys; Glasgow, Manchester and Liverpool City Council websites; and Google (using the search terms ‘Scotland’, ‘England’, ‘Diet’, ‘Glasgow’, ‘Manchester’, ‘Liverpool’).

The reference lists of included grey literature and database papers were also hand-searched. To limit the scope of the work, only those references that could be retrieved within the 4-month study identification period were included.

Two researchers scanned titles and abstracts independently to identify publications requiring full-text review. The project lead acted as a third reviewer when there were disagreements. Inclusion was determined by a single reviewer examining the full text of publications with support from the project lead.

### Data extraction

The Cochrane Collaboration^(^ ^19^ ^)^ data extraction form was adapted to make it more specific to the present review; for example, by removing items referring to experimental and quasi-experimental studies. Variables extracted included geographical area (e.g. country, region), study type (e.g. population-based observational study), survey name (e.g. Scottish Health Survey), study population (e.g. age, gender, socio-economic group), sample size, sampling method (e.g. random, convenience), survey administration (interviewer, mail, telephone, self-report), dietary assessment method (e.g. 24 h recall, weighed diary, FFQ), dietary outcome (e.g. fruit and vegetable intake, energy intake, vitamin intake), units of measurements (e.g. mg/d, portions/d), nutrient database used in the analysis, confounding variables (e.g. age, gender, socio-economic status, income, area deprivation) and dietary analysis software.

### Study quality

The Quality Assessment Tool for Observational Cohort and Cross-sectional Studies, developed by the National Institutes of Health/National Heart, Lung, and Blood Institute^(^ ^20^ ^)^, was used as a checklist for scoring study quality. The checklist assessed how representative the study population was, sample sizes, response rates, reliability and validity of measures, and adjustment for key confounders. An additional criterion was added as to whether the statistical analysis carried out in the study was suitable for answering the review questions. Studies were scored on a continuum of poor–fair–good quality dependent on individual scores for the criteria outlined above. Full information on study quality can be found in the online supplementary material, Supplemental Tables 5 and 6.

### Data synthesis

Data were synthesised by dietary outcomes. Comparisons that tested for statistical significance were examined in greatest depth, covering foods consumed, macronutrients and micronutrients. Data that provided information on dietary trends, but did not test for statistical significance, were then examined. Where information was available for an English region only rather than England as a whole, the most relevant region for the study objectives was chosen for comparison. We chose to compare Scottish data with Northern England as this region is demographically the most similar to Scotland. The review was focused on publications with information for both Scotland and England; however, the study team compared national surveys carried out separately in England and Scotland where outcomes had been measured similarly and around the same time period (e.g. difference of 2 years or less).

## Results

**Fig. 1:**
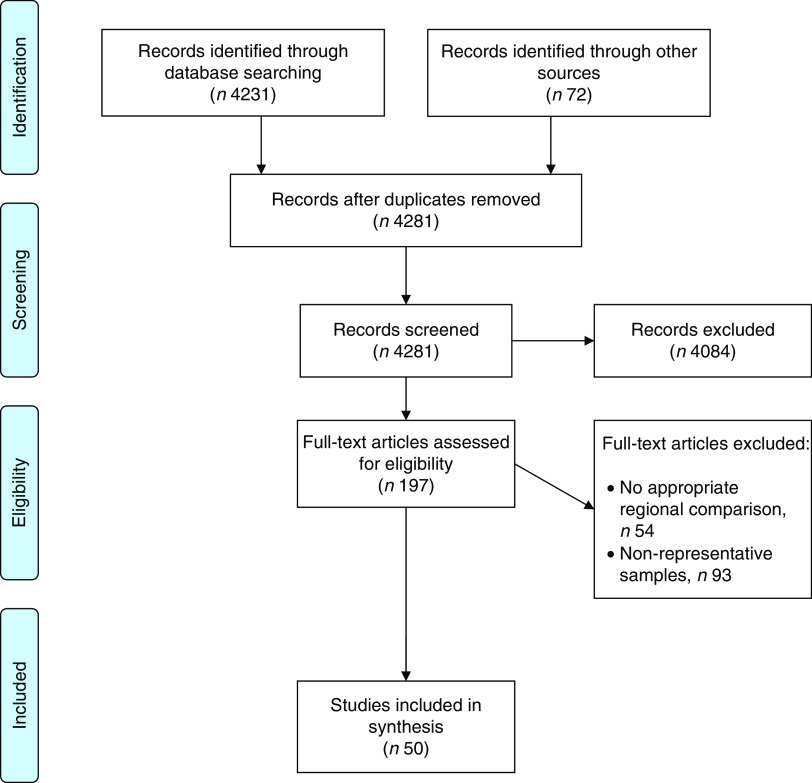
Flowchart showing the studies retrieved for the present rapid literature review on dietary differences between Scotland and England

Figure 1 details a flow diagram of the search results. The database searches returned 4231 results. The scoping, Internet and reference list searches identified seventy-two results. After removal of duplicates, titles and abstracts were screened for 4281 results with full-text examination of 197 publications. The team excluded 147 publications due to non-representative samples or no suitable comparison for Scotland and England. From the grey literature, we identified reports from a number of national surveys. These included the Health Survey for England^(^ ^21^ ^–^ ^24^ ^)^, the Scottish Health Survey^(^ ^25^ ^–^ ^29^ ^)^, the National Diet and Nutrition Survey and predecessors^(^ ^30^ ^–^ ^37^ ^)^, the National Food Survey/Expenditure and Food Survey/Living Costs and Food Survey^(^ ^38^ ^–^ ^54^ ^)^, and the Low Income Diet and Nutrition Survey^(^ ^55^ ^)^. A number of volumes from a single survey report were identified (e.g. Low Income Diet and Nutrition Survey; Scottish Health Survey). Multiple volumes for a survey in the same year were considered a single publication. Included publications provided data on a wide range of dietary outcomes (Tables 2 and 3). Three publications tested differences between Scotland and England as a whole statistically^(^ ^9^ ^,^ ^55^ ^,^ ^56^ ^)^, as opposed to English regions or England and Wales. A single publication^(^ ^56^ ^)^ statistically tested data at the regional level, comparing Greater Glasgow and the North West of England, and Greater Glasgow and Greater Manchester.

**Table 2:** Overview of child studies

Author and quality rating	Scotland	England	Response rate (%)	Dietary outcome	Data adjustment and statistical analysis
**1946 British Birth Cohort (4-year-old children) (1950)** Multistage cluster probability sample, 24 h recall diet records (mother/carer responding), data weighted					
Prynne *et al*.^(^ ^58^ ^)^ Good–fair	*n* 527	*n* 1119	86	Dietary phylloquinone intake (μg/d, μg/MJ) Dietary phylloquinone intake (μg/kg BW/d, % below 1 μg/kg BW/d)	Energy intake, body weight (*P* values adjusted for sex and social class, but means presented unadjusted) ANOVA
Prynne *et al*.^(^ ^57^ ^)^ Fair	*n* 527	*n* 1119	86	Children consuming food once (%), energy intake (MJ/d), macronutrient intake (g/d), micronutrient intake (mg/d)	Social class, gender, season, food between meals, whether record contained weekday/weekend day Multiple logistic regression
**Diets of British School Children (aged 10/11 and 14/15 years) (1983)** Multistage cluster probability sample, 7 d weighed diary (parent and child completed), data weighted; North England = Northern, North West and Yorkshire/Humberside					
Committee on Medical Aspects of Food Policy^(^ ^30^ ^)^ Fair	10/11 years: males, *n* 45; females, *n* 424 14/15 years: males, *n* 56; females, *n* 42	North England 10/11 years: males, *n* 260; females, *n* 262 14/15 years: males, *n* 145; females, *n* 129	75	Food and food groups (g/week), energy intake (kJ/d), fibre intake (g/d), macronutrient intake (g/d), micronutrient intake (mg/d or μg/d)	Data adjusted: limited to age group and gender Statistical test not specified
**1970 British Cohort Study (aged 16/17 years old) (1986)** Multistage cluster probability sample, 4 d weighed diary, data weighting unclear, significance testing					
Crawley^(^ ^59^ ^)^	Males: *n* 85 Females: *n* 133	England/Wales Males: *n* 573 Females: *n* 824	Unclear (only 34 % diaries usable)	Food and food groups (% respondents, g/d), energy intake (kcal/d, MJ/d), macronutrient intake (% energy/d), fibre (g/d), micronutrient intake (mg/d or μg/d)	Class, benefits receipt, housing, household size, no. of parents, education, mothers’ working status, microwave ownership, dieting, body size, smoking, takeaway consumption, eating out, feeding frequency, alcohol, television viewing, sports Unadjusted results presented Generalised linear model
**National Diet and Nutrition Survey (NDNS) ** **1**·**5–4**·**5-year-olds (1992/93)** Multistage cluster probability sample, 4 d weighed diaries in 1992/93 (mother/carer completed), data weighted; Scotland excluding islands; Northern England including North Yorkshire and Humberside and North West, Merseyside					
Prynne *et al*.^(^ ^58^ ^)^ Pooled data: 4-year-olds from NDNS 1992/93 & 1997 Fair	*n* 34	Northern England *n* 79	81	Dietary phylloquinone intake (μg/d, μg/MJ) Dietary phylloquinone intake (μg/kg BW/d, % below 1 μg/kg BW/d)	Energy intake, body weight (*P* values adjusted for sex and social class, but means presented unadjusted) ANOVA
Gregory *et al*.^(^ ^37^ ^)^ Fair	*n* 165	Northern England *n* 427	81	Food and food groups (g/week, % consumers), energy intake (kJ/d, kcal/d), fibre intake (g/d), macronutrient intake (g/d, % energy), micronutrient intake (mg/d or μg/d)	Unadjusted results presented from bivariate analysis Statistical methods unclear
Watt *et al*.^(^ ^60^ ^)^ Fair	*n* 188	Northern England *n* 466	81	% children meeting one or none of five dietary parameters (RNI for Fe, Zn, vitamin A, vitamin C, % energy from NMES)	Data not adjusted for Scotland/Northern England comparison ANOVA for region, but no comparison for Scotland/Northern England specifically
**4–18-year-olds (1997)** Multistage cluster probability sample, 7 d weighed diary (mother/carer completed), weighted data					
Gregory and Lowe^(^ ^36^ ^)^ Fair	Males: *n* 68 Females: *n* 69	Northern England Males: *n* 243 Females: *n* 217	64	Food and food groups (g/week, % consumers), energy intake (kJ/d), fibre intake (g/d), macronutrient intake (g/d, % energy), micronutrient intake (mg/d or μg/d)	Confounding variables unclear Multiple regression; however, unadjusted results presented from bivariate analysis only
**Health Behaviour in School-aged Children (11, 13, 15 year olds)** Stratified cluster probability sampling for some schools and classes, FFQ (not validated), data weighted					
Currie *et al*.^(^ ^61^ ^)^ (2001/2) Poor–fair	Males: *n* 2246 Females: *n* 2158 11-year-olds: *n* 1743 13-year-olds: *n* 1512 15-year-olds: *n* 1149	Males: *n* 2943 Females: *n* 3138 11-year-olds: *n* 2239 13-year-olds: *n* 2069 15-year-olds: *n* 1773	Unknown	Fruit daily Vegetables daily Soft drinks daily Sweets daily	Presented for age and gender Descriptive statistics
Currie *et al*.^(^ ^62^ ^)^ (2005/6)	Males: *n* 3032 Females: *n* 3113 11-year-olds: *n* 1691 13-year-olds: *n* 2256 15-year-olds: *n* 2198	Males: *n* 2308 Females: *n* 2460 11-year-olds: *n* 1655 13-year-olds: *n* 1662 15-year-olds: *n* 1451	Scotland: 65 England: unknown	Fruit daily Vegetables daily Soft drinks daily	As above
Currie *et al*.^(^ ^63^ ^)^ (2009/10)	Males: *n* 3319 Females: *n* 3419 11-year-olds: *n* 2055 13-year-olds, *n* 2116 15-year-olds: *n* 2567	Males: *n* 1522 Females: *n* 1981 11-year-olds: *n* 1185 13-year-olds: *n* 1200 15-year-olds: *n* 1118	England: 40 Scotland: 65	As above	As above
**Health Survey for England (children aged 5–15 years)** Multistage cluster probability sample, 24 h recall, data weighted					
Sproston and Primatesta^(^ ^21^ ^)^ (2003) Fair		Males: *n* 1350 Females: *n* 1285	73	Fruit & vegetable intake (portions/d)	Presented by gender Descriptive statistics
Craig *et al*.^(^ ^24^ ^)^ (2008)		Males: *n* 2640 Females: *n* 2514	Males: 62 Females: 63	As above	As above
Craig and Hirani^(^ ^22^ ^)^ (2009)		Males: *n* 1367 Females: *n* 1312	Males: 68 Females: 69	As above	Presented by age and gender Descriptive statistics
Craig and Mindell^(^ ^23^ ^)^ (2013)		Males: *n* 701 Females: *n* 716	62	As above	Presented by age, gender, IMD, income Descriptive statistics
**Scottish Health Survey (children aged 5–15 years) (comparable to English data)** Multistage cluster probability sample, 24 h recall, data weighted					
Bromley *et al*.^(^ ^29^ ^)^ (2003) Fair	Males: *n* 1152 Females: *n* 1170		77	Fruit & vegetable intake (portions/d)	Presented for gender, income, SIMD, NS-SEC Descriptive statistics
Corbett *et al*.^(^ ^27^ ^)^ (2008)	Males: *n* 591 Females: *n* 597		64	As above	Presented by gender Descriptive statistics
Corbett *et al*.^(^ ^28^ ^)^ (2009)	Males: *n* 923 Females: *n* 837		69	As above	Presented by gender, income, NS-SEC Descriptive statistics
Bromley *et al*.^(^ ^26^ ^)^ (2013)	Males: *n* 608 Females: *n* 554		74	As above	Presented by age and gender Descriptive statistics
**Low Income Diet and Nutrition Survey – low-income households (2007)** Multistage cluster probability sample, 24 h recall, data weighted					
Nelson *et al*.^(^ ^55^ ^)^ Fair	Males: *n* 39 Females: *n* 39	Males: *n* 289 Females: *n* 313	55	Food and food groups (g/d, % consuming), fruit & vegetables (daily portions, % consuming 5+ portions daily, energy intake (MJ/d, EAR), macronutrient intake (g/d), micronutrient intake (mg/d or μg/d, RNI)	Presented by gender and household type Significance testing but analysis strategy not reported
**EURO-URHIS 2 (youth survey 14–16-year-olds) (2010)** Unclear sampling strategy, FFQ, data unweighted					
EURO-URHIS 2^(^ ^64^ ^–^ ^66^ ^)^ Poor	Glasgow: *n* 296	Greater Manchester: *n* 1128 Merseyside: *n* 3466	Unknown	Regular fruit and vegetable or salad consumption (% participants)	Data unadjusted Descriptive statistics

BW, body weight; RNI, Recommended Nutrient Intake; NMES, non-milk extrinsic sugars; SIMD, Scottish Index of Multiple Deprivation; NS-SEC, National Statistics Socioeconomic Classification; EAR, Estimated Average Requirement; IMD, Index of Multiple Deprivation. Dates in parentheses represent the year(s) in which data were collected.

**Table 3:** Overview of adult studies

Author and quality rating	Scotland	England	Response rate (%)	Dietary outcome	Data adjustment and statistical analysis
**1946 British Birth Cohort Study/NSHD (1982)** Multistage cluster probability sample, 5 d prospective diary method (self-report) and 2d interviewer-assisted recall (39 % complete data) using household measures to estimate portion size, data weighting not reported; North England = North England, Yorkshire and Humberside					
Braddon *et al*.^(^ ^69^ ^)^ 36-year-old adults Fair	Men: *n* 120 Women: *n* 118	Northern England Men: *n* 290 Women: *n* 247	86	Energy (MJ), protein (g/d, % energy), fat (g/d, % energy), carbohydrate (g/d, % energy), alcohol (g/d), total fibre (g/d), cereal fibre (g/d), total sugars (g/d), added sugar (g/d), Fe (mg/d), Ca (mg/d), vitamin C (mg/d)	Not clear; possibly controlled for social class, education
**Health and Lifestyle Study (1984/5)** Multistage cluster probability sample, FFQ, self-report, data weighting not reported; Northern England = North West, Yorkshire and Humberside					
Whichelow *et al*.^(^ ^76^ ^)^ Fair	Adults: *n* 8860 Breakdown for regions unclear		73	Frequent consumption of food items (%) (fruit & vegetables, cereal products, meat and fish, dairy produce, miscellaneous)	Data unadjusted Descriptive statistics (statistical comparison only with South East England)
**Dietary and Nutritional Survey of British Adults (1987)** Multistage cluster probability sample, 7d weighed diary, self-report, data weighting not reported; Northern England = North, Yorkshire, Humberside and North West; Scotland = excluding islands					
Gregory *et al*.^(^ ^35^ ^)^ Fair–good	Men: *n* 96 Women: *n* 95	North England Men: *n* 274 Women: *n* 290	70	Food and food groups (g/d, % consuming), energy intake (kcal/d), macronutrient intake (g/d, % food energy from fat/d), micronutrient intake (mg/d or μg/d)	Energy intake, social class, economic status, unwell, slimming, cigarette smoking, drinking behaviour, health-related diet ANOVA; *F* test for overall regional comparison, no multiple comparison
**National Food Survey/Expenditure and Food Survey/Living Costs and Food Survey (household-level data)** Multistage cluster probability sample, food purchase data, self-report, data weighted					
MAFF^(^ ^53^ ^)^ (1997) Fair	Households: *n* 550 Individuals: *n* 1347	Households: *n* 5280; Individuals: *n* 12 822	65	Food and food groups (g/week, ml/week), energy intake (MJ/d, kcal/d), macronutrient intake (g/d), micronutrient intake (mg/d or μg/d)	Data unadjusted Descriptive statistics
MAFF^(^ ^54^ ^)^ (1998)	Households: *n* 541 Individuals: *n* 1341	Households: *n* 5073 Individuals: *n* 12 556	65	As above	As above
MAFF^(^ ^52^ ^)^ (1999)	Households: *n* 541 Individuals: *n* 1263	Households: *n* 5252 Individuals: *n* 12 969	6	As above	As above
MAFF^(^ ^51^ ^)^ (2000)	Households: *n* 548 Individuals: *n* 1320	Households: *n* 5097 Individuals: *n* 12 488	64	As above	As above
Defra^(^ ^44^ ^)^ (2001/2)	Households: *n* 622 Individuals: *n* 1431	Households: *n* 5965 Individuals: *n* 14 913	62	As above	As above
Defra^(^ ^43^ ^)^ (2002/3)	Households: *n* 585 Individuals: *n* 1346	Households: *n* 5400 Individuals: *n* 12 906	58	As above	As above
Defra^(^ ^45^ ^)^ (2003/4)	Households: *n* 585 Individuals: *n* 1340	Households: *n* 5626 Individuals: *n* 13 502	58	As above	As above
Defra^(^ ^39^ ^)^ (2004/5)	Households: *n* 1724 Individuals: *n* 3965	Households: *n* 4680 Individuals: *n* 16 240	57	As above	As above
Defra^(^ ^47^ ^)^ (2005/6)	Households: *n* 1706 Individuals: *n* 3924	Households: *n* 16 199 Individuals: *n* 38 878	57	As above	As above
Defra^(^ ^46^ ^)^ (2006)	Households: *n* 1589 Individuals: *n* 4450	Households: *n* 14 450 Individuals: *n* 34 680	55	As above	As above
Defra^(^ ^40^ ^)^ (2007)	Households: *n* 1499 Individuals: *n* 2698	Households: *n* 13 889 Individuals: *n* 33 334	53	As above	As above
Defra^(^ ^41^ ^)^ (2008)	Households: *n* 1583 Individuals: *n* 3482	Households: *n* 14 437 Individuals: *n* 34 649	53	As above	As above
Defra^(^ ^42^ ^)^ (2009)	Households: *n* 1545 Individuals: *n* 3389	Households: *n* 13 678 Individuals: *n* 32 827	51	As above	As above
Scarborough *et al*.^(^ ^9^ ^)^ (2007–2009) Fair–good	Combined sample from 2007, 2008, 2009 17 811 UK households, no regional breakdown provided (all household members over 7 years of age)		50–53	Energy (kcal/d); total, saturated, monounsaturated and polyunsaturated fats (g/d); cholesterol (mg/d); fibre (g/d); salt (g/d); fruit (g/week); vegetables (g/week)	DIETRON model^(^ ^92^ ^)^ uses number of deaths delayed or averted for Scotland based on age-, gender- and cause-specific mortality data
Defra^(^ ^50^ ^)^ (2010)	Households: *n* 1512 Individuals: *n* 3327	Households: *n* 13 300 Individuals: *n* 31 920	50	As in previous reports	Data unadjusted Descriptive statistics
Defra^(^ ^48^ ^)^ (2011)	Households: *n* 1512 Individuals: *n* 3629	Households: *n* 13 574 Individuals: *n* 33 935	54	As above	As above
Defra^(^ ^38^ ^)^ (2012)	Households: *n* 1451 Individuals: *n* 3482	Households: *n* 13 843 Individuals: *n* 34 608	52	As above	As above
Defra^(^ ^49^ ^)^ (2013)	Households: *n* 1395 Individuals: *n* 3069	Households: *n* 13 791 Individuals: *n* 33 098	48	As above	As above
**National Diet and Nutrition Survey 19–64 year olds (2001/2)** Multistage cluster probability sample, 7 d weighed record, dietary interview, 24 h urinary sample, self-report and objective (for urinary sample), data weighted					
Haleem *et al*.^(^ ^74^ ^)^ Fair	*n* 123	*n* 451	47	Antioxidant intake from fruit & vegetables (μmol/d)	Data unadjusted Descriptive statistics
Henderson *et al*.^(^ ^31^ ^–^ ^33^ ^)^ Fair	Dietary interview: men, *n* 80; women, *n* 111 Weighed record: men, *n* 53; women, *n* 70	Northern England Dietary interview: men, *n* 267; women, *n* 341 Weighed record: men, *n* 19; women, *n* 256	47	Food and food groups (g/week, % consumers), energy intake (kJ/d), fibre intake (g/d), macronutrient intake (g/d, % energy), micronutrient intake (mg/d or μg/d)	Data unadjusted Descriptive statistics
Ji *et al*.^(^ ^70^ ^)^ Fair	Men: *n* 53 Women: *n* 70	Northern England Men: *n* 195 Women: *n* 256	47	Na intake (mg/d) Urinary Na excretion (mmol/d)	Gender, smoking habit, social class, marital status, education, age, BMI, alcohol drinking, energy intake Bayesian geo-additive mixed models
**Sodium surveys – England and Scotland** Participants recruited via probability sample in Health Survey for England 2005, Scottish Health Surveys 2003 and 2008, National Diet and Nutrition Survey rolling programme, telephone interview and nurse visit, 24 h urinary Na, data weighted					
NatCen & UCL^(^ ^72^ ^)^ (2005/06) Poor–fair		Men: *n* 217 Women: *n* 313	20	Salt intake (g/d)	Presented by age and gender Descriptive statistics
NatCen & UCL^(^ ^71^ ^)^ (2003) Fair	Men: *n* 243 Women: *n* 331		32–34	Salt intake (g/d)	As above
ScotCen Social Research^(^ ^68^ ^)^ (2009) Poor–fair	Men: *n* 320 Women: *n* 382		Households: 58 Individuals: 50	Salt intake (g/d)	As above
Sadler *et al*.^(^ ^73^ ^)^ (2011) Poor–fair		Men: *n* 250 Women: *n* 297	24	Salt intake (g/d)	As above
**Low Income Diet and Nutrition Survey (2007)** Multistage cluster probability sample, 24 h recall, interviewer administered face-to-face, data weighted					
Nelson *et al*.^(^ ^55^ ^)^ Low-income individuals Fair	Men: *n* 120 Women: *n* 194	Men: *n* 609 Women: *n* 1222	55	Food and food groups (g/d, % consuming), fruit & vegetable intake (portions/d, % consuming 5+ portions/d), energy intake (MJ/d, EAR), macronutrient intake (g/d), micronutrient intake (mg/d or μg/d, and RNI)	Presented by gender and household type Significance testing but analysis strategy not reported
**Health Survey for England (HSfE)** Multistage cluster probability sample, 24 h recall, interviewer administered face-to-face, data weighted					
Shelton^(^ ^56^ ^)^ (2003) Good–fair	SHS 2003: *n* 8148	HSfE 2003: *n* 14836	HSfE: 66 SHS: 60	% eating 5+ fruit & vegetable portions/d OR for eating 5+fruit & vegetable portions/d	Socio-economic status, income, age, urban residence, smoking Logistic regression
Craig *et al*.^(^ ^24^ ^)^ (2008) Fair		Men: *n* 7325 Women: *n* 7682	Households: 64 Boost: 73	Fruit & vegetable intake (portions/d) % eating 5+fruit & vegetables daily	Presented by age, gender, NS-SEC, income Descriptive statistics
Craig and Hrani^(^ ^22^ ^)^ (2009)		Men: *n* 2107 Women: *n* 2537	Households: 68	As above	Presented by age, gender, income Descriptive statistics
Craig and Mindell^(^ ^23^ ^)^ (2013)		Men: *n* 3924 Women: *n* 4866	Households: 64 All adults: 58	As above	Presented by age, gender, IMD, income Descriptive statistics
**Scottish Health Survey (SHS)** Multistage cluster probability sample, 24 h recall, interviewer-administered face-to-face, data weighted					
Corbett *et al*.^(^ ^27^ ^)^ (2008) Fair	Men: *n* 2840 Women: *n* 3621		Households: 61	Fruit & vegetable intake (portions/d) % eating 5+fruit & vegetable portions/d	Presented by age, gender, income, SIMD Descriptive statistics
Corbett *et al*.^(^ ^28^ ^)^ (2009)	Men: *n* 3283 Women: *n* 4241		Households: 64	As above	Presented by age and gender Descriptive statistics
Bromley *et al*.^(^ ^26^ ^)^ (2013)	Men: *n* 2138 Women: *n* 2754		Households: 64	As above	As above

NSHD, National Survey of Health and Development; MAFF, Ministry of Agriculture, Fisheries and Food; Defra, Department for Environment, Food and Rural Affairs; NatCen, National Centre for Social Research; UCL, University College London; EAR, Estimated Average Requirement; RNI, Recommended Nutrient Intake; NS-SEC, National Statistics Socioeconomic Classification; IMD, Index of Multiple Deprivation; SIMD, Scottish Index of Multiple Deprivation. Dates in parentheses represent the year in which data were collected.

Results are presented separately for dietary differences between Scotland and England in children and adults. Tables 2 (children) and 3 (adults) present an overview of the methodology used in each study. Table 3 details studies on adults and includes publications from the National Food Survey/Expenditure and Food Survey/Living Costs and Food Survey^(^ ^38^ ^–^ ^54^ ^)^ where food purchase data were collected at the household level and analysed to estimate per person intakes of foods and nutrients. Information on child intake is included within the reports from the aforementioned surveys as part of the household sample; however, child-only results were not presented.

Results for those studies that carried out statistical tests of difference on data from Scotland and England are presented in Table 4 (children) and Table 5 (adults or households). Narrative results provide an overview of these studies, as well as referencing other studies that did not test for statistical difference, but support or contradict those studies that did.

**Table 4:** Significant results from studies with child populations


**British Birth Cohort (1950)** **Prynne** ***et al***.^(^ ^58^ ^)^ 4-year-olds: Scotland, *n* 527; Northern England, *n* 1119									
Dietary phylloquinone intake	Scotland	Northern England	*P*						
mg/d	27	35	<0·05						
μg/kg BW/d	1·6	2·1	<0·05						
% <1 μg/kg BW/d	33	26	<0·05						
**Prynne** ***et al***.^(^ ^57^ ^)^									
Child consumption of food groups									
at least once (%)	Scotland	Northern England	Adjusted OR	95 % CI	Daily intakes, adjusted means	Scotland	Northern England	*P*	
Porridge	30	11	0·3	0·2, 0·4	Energy (MJ)	5·7	6·2	<0·0001	
Cake, biscuits	52	60	1·4	1·1, 1·7	Carbohydrate (g)	164	187	<0·0001	
Eggs	64	51	0·6	0·5, 0·7	Sugar (g)	56	64	<0·0001	
Spreading fats	76	83	1·5	1·2, 1·9	Fat (g)	60	65	<0·0001	
Fried foods	30	43	1·8	1·4, 2·3	Fe (mg)	7·2	7·8	<0·0001	
Bacon	17	28	1·9	1·5, 2·5	Mg (mg)	160	171	<0·0001	
Vegetables	59	80	3·0	2·4, 3·8	K (mg)	1668	1769	0·006	
Fruit	34	41	1·4	1·1, 1·7	Carotene* (µg)	498	612	0·008	
Orange juice	6	9	1·6	1·1, 2·5	Thiamin (mg)	0·69	0·73	0·001	
Soup	36	4	0·1	0·0, 0·1	Vitamin C* (mg)	24	32	<0·0001	
**Diets of British School Children (1983)** **Committee on Medical Aspects of Food Policy** ^(^ ^30^ ^)^ Scotland: 10/11 years, males, *n* 457; females, *n* 424; 14/15 years, males, *n* 56; females, *n* 42; North England: 10/11 years, males, *n* 260; females, *n* 262; 14/15 years: males, *n* 145; *n* 129 females Significant differences discussed (but not given) for Scotland *v*. England/Wales only and at times unclear (Northern England figures presented below)									
	Males					Females			
	10/11-year-olds		14/15-year-olds			10/11-year-olds		14/15-year-olds	
	Scotland	Northern England	Scotland	Northern England		Scotland	Northern England	Scotland	Northern England
Vegetables (g/week)	469	568			Vegetables (g/week)	411	488		
Cakes (g/week)	121	159			Carrots (g/week)	49	54		
Biscuits (g/week)	159	200			Fruit juice (g/week)	406	333	348	253
Pudding (g/week)	354	500			Beef (g/week)			112	78
Other meat products (g/week)	247	395			Soup (g/week)			463	199
Potatoes (g/week)	453	423			Retinol (µg/d)	430	480	360	680
Beef (g/week)	165	82			Carotene (µg/d)	920	1390	920	1620
Milk (g/week)	2127	1851	2171	1837	Retinol equivalents (µg/d)	590	710	510	950
Cheese (g/week)	111	65	168	135	Thiamin (mg/d)	0·95	1·06		
Sausages (g/week)	146	119			Riboflavin (mg/d)	1·36	1·45		
Chocolate (g/week)	133	101			Nicotinic acid equivalents (mg/d)	22·6	23·6		
Sweets (g/week)	142	120			Vitamin D (µg/d)	1·15	1·36	1·09	1·33
Soup (g/week)	431	254			Pyridoxine (mg/d)	0·99	1·07		
Fat (g/d)	87·2	87·6			Vitamin C (mg/d)	40·6	44·1	43·1	44·8
Retinol (µg/d)	450	570			Fe (mg/d)			8·8	9·5
Carotene (µg/d)	1000	1430							
Retinol equivalents (µg/d)	620	810							
Vitamin D µ/d	1·24	1·54							
Pyridoxine (mg/d)	1·14	1·17							
Vitamin C (mg/d)	42·5	43·6							
**1970 British Cohort Study (1986)** **Crawley** ^(^ ^59^ ^)^ 16/17-year-olds; Scotland: males, *n* 85; females, *n* 133; England/Wales: males, *n* 573; females, *n* 824									
	Males				Females				
Nutrient intake	Scotland	England/Wales	*P*	Nutrient intake	Scotland	England/Wales	*P*		
NSP (g/d)	14·5	16·2	<0·001	NSP (g/d)	11·4	13·0	<0·001		
Mg (mg/d)	312	328	<0·01	Mg (mg/d)	247	262	<0·01		
Cu (mg/d)	1·56	1·64	<0·01	P (mg/d)	1068	1121	<0·01		
Retinol (µg/d)	979	1205	<0·001	Retinol (µg/d)	796	974	<0·001		
Carotene (µg/d)	1540	2122	<0·001	Carotene (µg/d)	1490	2032	<0·001		
Riboflavin (mg/d)	1·91	2·05	<0·01	Riboflavin (mg/d)	1·35	1·47	<0·001		
Vitamin B_6_ (mg/d)	2·07	2·13	<0·01						
Vitamin B_12_ (µg/d)	4·73	5·33	<0·01						
Folates (µg/d)	299	314	<0·01						
Food group intake	Males			Food group intake	Females				
(% consuming or intake g/d)	Scotland	England/Wales	*P*	(% consuming or intake g/d)	Scotland	England/Wales	*P*		
Beer (%)	28	37	<0·01	Beer (%)	13	25	<0·01		
Fizzy drinks (not low-calorie) (%)	91	75	<0·001	Beer intake (g/d)	240	190	<0·01		
Squash (g/d)	152	219	<0·01	Hot chocolate (%)	13	24	<0·01		
Hot chocolate (%)	11	22	<0·01	White bread (g/d)	62	91	<0·01		
All bread (g/d)	127	99	<0·01	Breakfast cereals (g/d)	14	64	<0·01		
White bread (g/d)	99	93	<0·01	Skimmed milk (%)	4	14	<0·01		
Pasta and rice (%)	37	55	<0·01	Polyunsaturated fat spreads (%)	10	21	<0·01		
Skimmed milk (%)	4	9	<0·01	Non-fried potatoes (%)	23	44	<0·001		
Non-fried potatoes (%)	34	50	<0·01	Chips (g/d)	97	71	<0·001		
Chips (g/d)	121	101	<0·001	All veg (%)	94	97	<0·001		
All veg (%)	80	93	<0·001	All veg (g/d)	56	83			
All veg (g/d)	57	84		Green veg (g/d)	26	50	<0·001		
Green veg (g/d)	22	46	<0·001	Carrots (g/d)	24	47	<0·001		
Carrots (g/d)	19	48	<0·01						
**National Diet and Nutrition Survey (NDNS)**									
**Watt** ***et al***.^(^ ^60^ ^)^ **(1993)** 1·5–4·5-year-olds: Scotland, *n* 188; Northern England, *n* 466									
	Scotland	Northern England	*P*						
% children meeting one or none of 5 dietary parameters	54	49	<0·001 (for overall regional comparison only)

**Table 5:** Significant results from studies with adult populations


**1946 British Birth Cohort Study/NSHD (1982)** Braddon *et al*.^(^ ^69^ ^)^ 36-year-olds; Scotland: men, *n* 120; women, *n* 118; North England: men, *n* 290; women, *n* 247							
	Males (mean values)				Females (mean values)		
Intake	Scotland	Northern England	*P*	Intake	Scotland	Northern England	*P*
Energy (MJ/d)	10·0	10·4	<0·01	Fat (g/d)	78·9	75·3	<0·01
Fat (g/d)	102·4	105·6	<0·01	Total fibre (g/d)	15·1	14·0	<0·001
Carbohydrate (g/d)	261	269	<0·001	Fe (mg/d)	10·0	9·9	<0·01
Total fibre (g/d)	18·1	19	<0·01	Ca (mg/d)	727	722	<0·001
Cereal fibre (g/d)	8·1	8·9	<0·01	Vitamin C (mg/d)	63·0	47·6	<0·01
Added sugar (g/d)	71·1	80·0	<0·01				
Calcium (mg/d)	904	941	<0·01				
Vitamin C (mg/d)	57·0	57·0	<0·001				
**Dietary and Nutritional Survey of British Adults (1987)**							
Gregory *et al*.^(^ ^35^ ^)^ Scotland: men, *n* 96; women, *n* 95; North England: men, *n* 274; women, *n* 290							
	Males (deviation from grand mean)†				Females (deviation from grand mean)†		
Intake	Scotland	Northern England	*P*	Intake	Scotland	Northern England	*P*
Energy (kcal/d)	−159	22	<0·05	Fibre (g/d)	−0·9	−0·3	<0·05
Energy (kcal/d)*	−149	10	<0·05	Na (mg/d)	203	57	<0·01
Sugars (g/d)	−7·7	−2·8	<0·05	Na (mg/d)*	211	50	<0·01
Fibre (g/d)	−1·6	0·0	<0·01	Vitamin B_6_ (mg/d)	−0·87	−0·80	<0·05
Fibre (g/d)*	−1·6	0·3	<0·01	Vitamin B_6_ (mg/d)*	−0·98	−0·68	<0·05
Fat (g/d)	0·0	−2·10	<0·05				
Fat (% food energy/d)	0·47	−0·29	<0·05				
Saturated fat (% food energy/d)	0·36	−0·36	<0·01				
Fat (g/d)*	0·80	−1·4	<0·05				
Fat (% food energy/d)*	0·64	−0·35	<0·05				
Saturated fat (% food energy/d)*	0·5	−0·40	<0·01				
Ca (mg/d)	21	−46	<0·01				
Na (mg/d)	188	72	<0·01				
Ca (mg/d)*	14	−46	<0·01				
Na (mg/d)*	198	74	<0·01				
Vitamin B_6_ (mg/d)	0·11	0·25	<0·05				
Folate (μg/d)	−9·4	13·1	<0·05				
Vitamin B_6_ (mg/d)*	0·19	0·18	<0·05				
Folate (μg/d)*	−12·8	8·1	<0·05				
**National Diet and Nutrition Survey 19–64 year olds (2000/1)**							
Ji *et al*.^(^ ^70^ ^)^ Scotland: men, *n* 53; women, *n* 70; Northern England: men, *n* 195; women, *n* 256 Results presented diagrammatically only. Results showed statistically significant positive spatial effect for respondents in Scotland compared with the posterior mean for dietary Na intake and urinary Na intake. No significant differences from the posterior mean were observed for respondents from Northern England. Highest UK levels of dietary Na intake and urinary Na were present in Scotland. Second highest levels were observed in Northern England							
**Health Survey for England (2003) & Scottish Health Survey (2003)** Shelton^(^ ^56^ ^)^ Scottish Health Survey 2003: *n* 8148; Health Survey for England 2003: *n* 14 836							
	% population				Odds of Manchester respondents eating 5+ portions of fruit & vegetables/d *v*. Glasgow respondents (controlling for NS-SEC, equivalised income and age)		
	Males		Females		Males		Females
	Scotland	England	Scotland	England	OR	95 % CI	
Eating 5+ fruit & veg/d	20	22	22	26	0·67	0·47, 0·94	Not significant
**Low Income Diet and Nutrition Survey (2007)** Nelson *et al*.^(^ ^55^ ^)^ Low-income households; Scotland: men, *n* 120; women, *n* 194; England: men, *n* 609; women, *n* 1222 No significant levels reported							
	Men				Women		
Intake	Scotland	England	Intake		Scotland	England	
Fruit (portions/d) (% consuming 5+portions/d)	1·9 (2)	2·5 (9)	Fibre (NSP) (g/d) (% consuming RDM)		9·7 (16)	10·9 (33)	
Na (mg/d)	3250	2872	Fruit (portions/d) (% consuming 5+portions/d)		1·9 (5)	2·6 (10)	
Vitamin D (median % RNI)	2·50		Na (mg/d)		2304	2075	
Vitamin A (median % RNI)	23	29	Vitamin A (median % RNI)		85	101	
**National Food Survey/Expenditure and Food Survey/Living Costs and Food Survey (2007–2009)**							
Scarborough *et al*.^(^ ^9^ ^)^ 17 811 UK households (all household members over 7 years of age) Statistical analysis: 40 % (95 % CI 33, 51 %) of the morality gap would be closed if Scottish population ate a diet in line with the English population							
Intake	Scotland	England					
Energy (kcal/d)	2375	2282					
Total fat (g/d)	98·1	94·6					
Saturated FA (g/d)	37·8	35·7					
MUFA (g/d)	36·3	35·3					
PUFA (g/d)	17·5	17·3					
Cholesterol (mg/d)	268	265					
Fibre (g/d)	15·0	15·1					
Salt (g/d)	7·5	7·0					
Fruit (g/week)	1205	1270					
Vegetables (g/week)	951	1190					

NSHD, National Survey of Health and Development; NS-SEC, National Statistics Socioeconomic Classification; RDM, recommended daily minimum; RNI, Recommended Nutrient Intake. Dates in parentheses represent the year in which data were collected.

* Adjusted for behavioural variables: cigarette smoking, food supplements, drinking behaviour, health-related diet.

† The grand mean is the mean of the means for each regional sub-sample.

### Children and young people

Seven studies identified statistically significant differences in the diets of children living in Scotland and England. The main findings from these data were that dietary intake in Scotland appeared to be lower in nutritional quality than that in England or Northern England. Dietary differences were present as early as 1950, although not always negatively for Scotland. Prynne *et al*.^(^ ^57^ ^)^ found that 4-year-old children living in Scotland had lower intakes of vegetables and fruit; however, they also found positive differences, with higher intakes of porridge and soup, and lower intakes of cakes, biscuits, fried foods and bacon. Energy intakes were lower in children living in Scotland in 1950, which perhaps explains why macro- and micronutrient intakes were also lower^(^ ^57^ ^,^ ^58^ ^)^. Lower micronutrient intake was identified in Scotland for a number of vitamins and minerals in three additional studies^(^ ^30^ ^,^ ^59^ ^,^ ^60^ ^)^. In contrast, in the 2007 Low Income Diet and Nutrition Study^(^ ^55^ ^)^, boys living in low-income households in Scotland were less likely than those living in England to have intakes of Ca, K and Zn below the Low Nutrient Reference Intakes. No other studies provided data on significant differences in macronutrient intakes; however, intakes of fibre were lower for 16- and 17-year-olds living in Scotland in 1986^(^ ^59^ ^)^. Differences in food consumption were identified and included children in Scotland being less likely to consume vegetables^(^ ^30^ ^,^ ^57^ ^,^ ^59^ ^,^ ^60^ ^)^, fruit^(^ ^55^ ^,^ ^57^ ^)^, spreading fat^(^ ^57^ ^)^, skimmed milk^(^ ^59^ ^)^, breakfast cereals^(^ ^36^ ^,^ ^59^ ^)^ and cakes^(^ ^30^ ^,^ ^57^ ^)^. Children in Scotland were more likely to consume chips^(^ ^59^ ^)^ and soup^(^ ^30^ ^,^ ^57^ ^)^. Consistent findings were identified only for soup, cake, vegetable and fruit consumption.

Similar results were reflected in studies that did not test for statistical differences in relation to lower fruit and vegetable intakes^(^ ^22^ ^–^ ^24^ ^,^ ^26^ ^–^ ^28^ ^)^. In the Health Behaviour in School-aged Children surveys a higher percentage of children in Scotland reported eating fruit daily in 2001/2^(^ ^61^ ^)^, but this pattern was demonstrated only for 11-year-olds, not 13- and 15-year-olds in 2005/6^(^ ^62^ ^)^ and 2009/10^(^ ^63^ ^)^. In line with the patterns identified in other studies, a lower percentage of children in Scotland reported eating vegetables daily in 2005/6^(^ ^62^ ^)^ and 2009/10^(^ ^63^ ^)^, and a higher percentage of children living in Scotland reported drinking sweetened beverages daily in 2001/2^(^ ^61^ ^)^ and 2005/6^(^ ^62^ ^)^. By 2009/10, a higher proportion of English children reported drinking them daily^(^ ^63^ ^)^. The EURO-URHIS 2 study found little difference in regular fruit and vegetable consumption between teenagers living in Glasgow and Greater Manchester; however, consumption was higher in Merseyside^(^ ^64^ ^–^ ^66^ ^)^.

In the only study to examine overall diet quality as a single variable, Watt *et al*.^(^ ^60^ ^)^ analysed the percentage of pre-school children meeting five dietary parameters (recommended nutrient intakes for Fe, Zn, vitamins A and C, and percentage of energy from non-milk extrinsic sugars of less than 10 %). Their analysis showed that children living in Scotland were less likely to meet one or more of the recommended dietary parameters.

#### Study quality

Overall study quality ranged from ‘poor’ to ‘good–fair’. The main limitations of the studies were that sample sizes were often small in Scotland^(^ ^11^ ^,^ ^21^ ^,^ ^45^ ^–^ ^47^ ^,^ ^52^ ^,^ ^53^ ^,^ ^67^ ^,^ ^68^ ^)^, limiting the ability to find statistically significant differences, and results often did not adjust for confounders^(^ ^21^ ^–^ ^24^ ^,^ ^26^ ^–^ ^30^ ^,^ ^36^ ^,^ ^37^ ^,^ ^55^ ^,^ ^61^ ^–^ ^66^ ^)^ or descriptive results were presented only^(^ ^21^ ^–^ ^24^ ^,^ ^26^ ^–^ ^30^ ^,^ ^36^ ^,^ ^37^ ^,^ ^58^ ^,^ ^60^ ^–^ ^63^ ^)^. The validity of dietary measures was less problematic. Only two surveys (with results reported across six reports)^(^ ^61^ ^–^ ^66^ ^)^ used a food frequency measure with no information on whether these measures had been validated. The remaining studies used either weighed 4d or 7d diaries^(^ ^30^ ^,^ ^36^ ^,^ ^37^ ^,^ ^58^ ^–^ ^60^ ^)^ or interviewer-assisted 24 h recalls^(^ ^21^ ^–^ ^24^ ^,^ ^26^ ^–^ ^29^ ^,^ ^55^ ^,^ ^57^ ^,^ ^58^ ^)^.

### Adults

Six studies presented statistically significant findings of differences between the diets of adults living in Scotland and adults living in England. Differences in energy intake appeared to vary by gender. For example, Gregory *et al*.^(^ ^35^ ^)^ reported that energy intake for men in Scotland was 210 kcal/d (879 kJ/d) lower compared with men in the North of England, but no differences were reported for women. Braddon *et al*.^(^ ^69^ ^)^ found a similar result, with men living in Scotland consuming 0·4 MJ/d less than men living in Northern England. However, women living in Scotland in the same survey had higher energy intakes (0·3 MJ/d) than women in England.

There were no notable significant differences in macronutrient intakes, other than fibre. Fibre intake (NSP) was 1·2 g/d lower in women living in low-income households in Scotland compared with similar women in England^(^ ^55^ ^)^ and 17 % fewer women in Scotland achieved the recommended daily minimum compared with women in England. More mixed results for fibre were found by Braddon *et al*.^(^ ^69^ ^)^, with men in Scotland reporting lower fibre intakes than those in Northern England, but women in Scotland reporting higher intakes than women in Northern England.

Few studies reported statistically significant differences in micronutrient intakes. Na intake was higher in Scotland than in English regions for men^(^ ^35^ ^)^ in 1987 (dietary intake), with this difference still present in 2001^(^ ^70^ ^)^ (dietary intake and urinary Na). The four dietary sodium surveys^(^ ^68^ ^,^ ^71^ ^–^ ^73^ ^)^ (adults 19–64 years) undertaken in Scotland and England between 2006 and 2011 found that salt intake (measured from urinary Na levels) was 0·5 g/d higher in men in Scotland in 2006 compared with men in England. Salt intakes were higher for both men (0·7 g/d) and women (0·9 g/d) living in Scotland in 2009 compared with those in England in 2011. Although these differences were not tested for significance, they indicate that earlier differences have remained.

For other micronutrients, consistent trends were identified, such as lower intakes in Scotland for vitamins A, C, D and E; however, these differences have narrowed^(^ ^38^ ^–^ ^54^ ^)^ over time. In contrast, Haleem *et al*. found that antioxidant intake in Scotland was higher than that in Northern England, particularly among men^(^ ^74^ ^)^.

The most consistent differences for food consumption were for fruit and vegetable intake. Shelton^(^ ^56^ ^)^ found that consumption of five or more portions of fruit and vegetables daily was lower in Scotland for men and women than in England. No significant differences were identified; however, the odds of eating five or more portions daily for men or women was greater in Cheshire and Merseyside compared with Greater Glasgow. In Greater Manchester men were less likely to eat five portions of fruit and vegetables each day compared with Greater Glasgow. Consistent findings were reported, although not compared statistically, in the 2008 Scottish Health Survey and the Health Survey for England^(^ ^75^ ^)^. Respondents from England ate an average of half a portion more of fruit and vegetables daily than those in Scotland, and a lower percentage of respondents in Scotland reported eating five or more portions of fruit and vegetables daily (men: difference=5·1 %, 95 % CI 2·8, 7·4 %; women: difference=5·2 %, 95 % CI 3·1, 7·3 %). Similar findings were identified in a range of the included studies^(^ ^21^ ^–^ ^28^ ^,^ ^35^ ^,^ ^38^ ^–^ ^55^ ^,^ ^76^ ^)^. Other differences noted were higher intakes of processed potatoes and meat, and soft drinks in Scotland^(^ ^21^ ^–^ ^28^ ^,^ ^35^ ^,^ ^38^ ^–^ ^55^ ^,^ ^76^ ^)^. In England intakes of carcass meat^(^ ^38^ ^–^ ^54^ ^,^ ^63^ ^)^ and fresh potatoes^(^ ^52^ ^–^ ^54^ ^,^ ^63^ ^)^ were higher. Reported confectionery intake has been higher in Scotland compared with England in more recent years^(^ ^38^ ^,^ ^49^ ^,^ ^55^ ^)^.

#### Study quality

Studies with adult populations had similar issues with study quality as those reported for children: low sample sizes in Scotland^(^ ^31^ ^,^ ^32^ ^,^ ^33^ ^,^ ^35^ ^,^ ^55^ ^,^ ^68^ ^–^ ^70^ ^,^ ^74^ ^)^, lack of adjustment for confounders^(^ ^22^ ^–^ ^24^ ^,^ ^26^ ^–^ ^28^ ^,^ ^31^ ^–^ ^33^ ^,^ ^38^ ^–^ ^55^ ^,^ ^68^ ^,^ ^69^ ^,^ ^71^ ^–^ ^74^ ^,^ ^76^ ^)^ and limited statistical analysis^(^ ^6^ ^,^ ^7^ ^,^ ^13^ ^–^ ^17^ ^,^ ^22^ ^–^ ^24^ ^,^ ^27^ ^–^ ^30^ ^,^ ^32^ ^,^ ^34^ ^–^ ^44^ ^,^ ^54^ ^,^ ^57^ ^–^ ^59^ ^,^ ^68^ ^,^ ^73^ ^,^ ^76^ ^,^ ^77^ ^)^. The largest sample sizes were in reports from the Scottish Health Survey^(^ ^26^ ^–^ ^28^ ^)^, the Health Survey for England^(^ ^22^ ^–^ ^24^ ^)^, and the National Food Survey/Expenditure and Food Survey/Living Costs and Food Survey^(^ ^38^ ^–^ ^54^ ^)^, which had no statistical testing of differences between the two countries and, in the case of the latter, were reliant on household rather than individual-level data. Response rates were also relatively low for a number of studies^(^ ^68^ ^,^ ^70^ ^,^ ^72^ ^–^ ^74^ ^)^ and although the data were weighted to account for this on key demographic variables, it increases the likelihood of bias in the results.

## Discussion

The current rapid review study was a response to NHS Health Scotland’s request for an overview of the evidence on whether aspects of diet and nutrition differ, or have differed historically, between Scottish and English populations. Examining the current and historical differences in food and nutrient intakes in Scotland and England, we identified for Scotland lower intakes of fruit and vegetables, fibre and vitamins, and higher intakes of salt. Differences in fruit and vegetable intake appear to have persisted over time, as have differences in micronutrient intakes. There were few other consistent differences in food consumption over time that could be identified from the included studies.

What is clear is that dietary differences between Scotland and England are apparent from the early years, as demonstrated in the literature reporting on surveys of pre-school children from 1950 and 1992^(^ ^58^ ^)^, and appear to continue throughout adolescence^(^ ^30^ ^)^ and into adulthood^(^ ^9^ ^,^ ^56^ ^)^. In line with evidence that suggests that eating habits are established in childhood^(^ ^78^ ^,^ ^79^ ^)^, our results indicate that in Scotland nutritional disparities with England begin in the early years and persist. The impact of nutritional deficiencies, such as lower fruit and vegetable consumption, was highlighted by Oyebode *et al*.^(^ ^80^ ^)^ in an analysis of Health Survey for England data. Higher fruit and vegetable consumption was associated with lower likelihood of all-cause, cancer and cardiovascular mortality. The lowest mortality risk from any cause was identified for those eating seven or more portions of fruit and vegetables daily, with consumption of vegetables, salad and fresh or dried fruit associated with decreased mortality. Similar results were found in an Australian study, which again highlighted the protective effect of seven or more daily portions of fruit and vegetables on all-cause mortality^(^ ^81^ ^)^. A systematic review and meta-analysis of 142 prospective studies concluded that the greatest protective effect for all-cause mortality and CVD resulted from consumption of ten portions of fruit and vegetables per day^(^ ^82^ ^)^.

There was little evidence to determine whether there were differences in dietary intake in the cities of and/or regions surrounding Glasgow, Liverpool and Manchester. These cities have been used as exemplars in demonstrating the inequalities in mortality outcomes that exist between Scotland and England^(^ ^5^ ^)^.

Only three studies tested differences for Scotland and England statistically at a national level^(^ ^9^ ^,^ ^55^ ^,^ ^56^ ^)^. The majority of studies reported data from Northern England. As Northern England has a more similar demographic profile to Scotland than southern English regions, it is likely to minimise the dietary differences that exist at a national level. The same is true of studies reporting data from England and Wales jointly. Caution is urged also as the dietary data (except for the sodium surveys which measured Na excretion in urine) are self-reported or reported by parents and carers of children, and prone to reporting bias. It is recognised, for example, that obese adults tend to under-report energy intake^(^ ^83^ ^,^ ^84^ ^)^. Reported energy intake tended to be lower in Scotland, but it is unclear whether this reflects lower intakes or a greater tendency to under-report within Scotland. For example, Scotland has not reported lower levels of overweight and obesity, which would be the expected outcome of lower energy intakes^(^ ^75^ ^)^. At a population level there are not yet objective measures of dietary intake that can be utilised^(^ ^85^ ^,^ ^86^ ^)^; however, we would not expect dietary assessment data collected in Scotland to be less accurate than those collected in England. Weighed intake (considered the gold standard) was used in many of the studies, with only a small number using non-validated food frequency measures. Nevertheless, under- and misreporting are still likely to occur even with gold standard measures^(^ ^87^ ^)^. Additional limitations identified were relatively limited statistical analysis and a failure to adjust for key confounders.

### Study limitations

The main limitation of the current rapid review was that a more extensive search of additional databases and grey literature was not possible due to commissioners’ time constraints. Within the scope of the rapid review, the team took the decision to limit reports to those that were easily accessible within the limited review period of 4 months. We therefore did not include historical reports from the National Food Survey which began in 1940. Our first included report from this survey is from 1997, meaning that over 50 years of evidence on food expenditure was not included. These reports provided descriptive data only, with no adjustment for confounding factors. We therefore believe that these reports would not have altered the main findings of the review, which focused more on studies that tested for statistical differences between Scotland and England.

The review did identify a number of large-scale studies, such as the National Diet and Nutrition Survey, the Health Survey for England and the Scottish Health Survey, which provide data from representative samples in these regions. In recognition of the need to increase Scottish samples in national surveys, the National Diet and Nutrition Survey rolling programme included a Scottish boost sample for 2008/9 to 2011/12. The report from these data was excluded from the review as it only compared results from respondents living in Scotland with respondents from the full UK sample rather than England specifically. Differences identified included lower energy intakes in women aged 19–64 years living in Scotland and, in line with the review findings, lower intakes of fibre and vegetables across age groups and gender among respondents living in Scotland^(^ ^88^ ^)^. Given the cost required, and the interest in academic and policy groups of undertaking robust dietary surveys, it is likely that the review identified all relevant studies. One exception to this is food purchase market analysis data such as those collected by Kantar Worldpanel UK. Scottish data have only recently been published and were outside the time frame of the original review; however, there is no equivalent report available on English data^(^ ^67^ ^)^.

An additional limitation was that only a single reviewer decided on study inclusion after reading the full text and a single reviewer extracted data. There is a possibility that bias may have been introduced into the information selected due to this compromise^(^ ^89^ ^)^. We believe that the risk was minimised through a clear protocol agreed by all authors before the review took place. In addition, the project lead provided an additional opinion on any areas of uncertainty.

## Conclusions

There were limited comparisons of dietary intake between Scotland and England in the published literature and only two studies that allowed for comparisons at more local levels. In general, there were lower intakes of fruit and vegetables, vitamins and fibre in Scotland compared with England. Increasing fruit, vegetable and fibre intakes are key targets within the Scottish Dietary Goals, and the review results suggest that both adults and children need to be encouraged through policy action and implementation to improve in these areas. Review results were limited by small sample sizes for Scotland and limited adjustment for confounding factors. It is recognised that dietary quality is poorer in populations experiencing higher levels of deprivation^(^ ^90^ ^,^ ^91^ ^)^. In addition, dietary differences exist with regard to age and gender^(^ ^31^ ^,^ ^32^ ^,^ ^33^ ^,^ ^36^ ^,^ ^37^ ^)^. It is essential, therefore, that comparisons between Scotland and England are examined using large representative samples, with data that have been collected robustly and allow for confounders to be taken into account. Such work is necessary to provide insight into the potential causes of excess mortality in Scotland compared with England and to contribute to policy recommendations to address these inequalities.

## Supplementary material

For supplementary material accompanying this paper visit https://doi.org/10.1017/S1368980017001380. click here to view supplementary material
