# Supplementary material for: Identifying dietary differences between Scotland and England: a rapid review of the literature
Source: Public Health Nutr. 2017 Jul 20;20(14):2459–77. doi: 10.1017/S1368980017001380 (PMC10261349; doi:10.1017/S1368980017001380)
Supplement: Supplementary file 1 [file S1368980017001380sup001.docx]

### Online supplementary material

| **Supplemental Table 1 – Medline Search** | |
| --- | --- |
| 1 | (scotland or england or glasgow or manchester or liverpool or merseyside or strathclyde or lancashire).ab,ti. or Scotland/ or England/ |
| 2 | diet/ or energy intake/ or portion size/ or serving size/ or nutritional status/ or  dietary fats/ or fats, unsaturated/ or Diet Surveys/ or food habits/ or food preferences/ or Nutrition Surveys/ or food/ or dietary carbohydrates/ or dietary fiber/ or dietary proteins/ or dietary supplements/ or fast foods/ or fruit/ or micronutrients/ or trace elements/ or vitamins/ or vitamin d/ or vegetables/ or Sodium Chloride, Dietary/ or carbohydrates/ or dietary sucrose/ or (diet$ or nutrient$ or nutrition$ or energy intake or fruit$ or vegetable$ or vitamin$ or sugar or NMES or salt or sodium or eating habits).ab,ti. |
| 3 | (longitudinal or cohort or observational or cross-section$ or survey$ or questionnaire$).mp. or epidemiologic studies/ or cohort studies/ or longitudinal studies/ or follow-up studies/ or "national longitudinal study of adolescent health"/ or prospective studies/ or retrospective studies/ or cross-sectional studies/ |
| 4 | 1 and 2 and 3 |

###

| **Supplemental Table 2 – Embase Search** | |
| --- | --- |
| 1 | (scotland or england or glasgow or manchester or liverpool or merseyside or strathclyde or lancashire).ab,ti. |
| 2 | diet/ or caloric intake/ or fat/exp portion size/ or nutrition/ or child nutrition/ or dietary intake/ or food/ or food intake/ or maternal nutrition/ or nutrient/ or nutritional health/ or dietary fiber/ or diet supplementation/ or fast food/ or nutritional value/ or fruit/ or trace element/ or vitamin intake/ or vitamin D/ or vitamin supplementation/ or vitamin/ or vegetable/ or sodium chloride/ or carbohydrate/ or sucrose/ or eating habit/ or (diet$ or nutrient$ or nutrition$ or energy intake or fruit$ or vegetable$ or vitamin$ or sugar or NMES or salt or sodium or eating habits).ab,ti. |
| 3 | (longitudinal or cohort or observational or cross-section$ or survey$ or questionnaire$).mp. or longitudinal study/ or observational study/ or epidemiology/ or cross-sectional study/ or health survey/ or prospective study/ retrospective study/ |
| 4 | 1 and 2 and 3 |

| **Supplemental Table 3 – Cinahl Search** | |
| --- | --- |
| 1 | (MM "Scotland") OR TI Scotland OR AB Scotland OR (MM "England") OR TI england OR AB England OR TI Manchester OR AB Manchester OR TI liverpool OR AB Liverpool OR TI Glasgow OR AB Glasgow OR TI strathclyde OR AB strathclyde OR TI Merseyside OR AB Merseyside OR TI lancashire OR AB lancashire |
| 2 | MH "Diet+/SN/TD" OR MM "Portion Size" OR (MM "Food") OR (MM "Food Habits") OR (MM "Food Intake") OR (MM "Energy Density") OR (MM "Energy Intake") OR (MM "Nutrition") OR (MM "Adolescent Nutrition") OR (MM "Child Nutrition") OR (MM "Infant Nutrition") OR (MM "Nutrient Density") OR (MM "Dietary Carbohydrates") OR (MM "Dietary Fats") OR (MM "Dietary Fiber") OR (MM "Dietary Proteins") OR (MM "Dietary Sucrose") OR (MM "Dietary Supplements") OR (MM "Sodium Chloride, Dietary") OR (MM "Sodium, Dietary") OR (MM "Fruit") OR (MM "Vegetables") OR TI diet* OR AB diet* OR TI nutrient* OR AB nutrient* OR TI nutrition* OR AB nutrition* OR TI energy intake OR AB energy intake OR TI fruit* OR AB fruit* OR TI vegetable* or AB vegetable* or TI sugar or AB sugar or TI NMES or AB NMES or TI salt or AB salt or TI sodium or AB sodium or TI eating habits or AB eating habits |
| 3 | Exclude Medline |
| 4 | 1 and 2 and 3 |

| **Supplemental Table 4 – Web of Science Search** | |
| --- | --- |
| 1 | TITLE: (scotland or england or glasgow or manchester or liverpool or merseyside or strathclyde or lancashire) |
| 2 | TITLE: (diet$ or nutrient$ or nutrition$ or "energy intake" or fruit$ or vegetable$ or vitamin$ or sugar or NMES or salt or sodium or "eating habits" or carbohydrate* or fibre or fiber or fat*) |
| 3 | TOPIC: (longitudinal or cohort or observational or cross-section$ or survey$ or questionnaire$ or cohort or prospective or retrospective) |
| 4 | 1 and 2 and 3 |

| **Supplemental Table 5 – Quality Assessment – Child Studies** | | | | | | | | | | |
| --- | --- | --- | --- | --- | --- | --- | --- | --- | --- | --- |
| **Author** | **Overall rating** | **RQ not clear** | **Population not clear** | **Response rate <50%** | **Recruitment not standardised** | **Sample size not justified/ small** | **Independent variable not valid** | **Dependent variable not valid** | **No cofounding adjustment** | **Limited statistical analysis** |
| Prynne et al.^(58)^ | Good-Fair |  |  |  |  |  |  |  |  |  |
| Prynne et al.^(57)^ | Fair |  |  |  |  | x |  |  |  | x |
| Committee on Medical Aspects of Food^(30)^ | Fair |  |  |  |  |  |  |  | x | x |
| Crawley^(59)^ | Fair |  |  | Not Reported |  | x |  |  |  |  |
| Gregory et al.^(37)^ | Fair |  |  |  |  | x |  |  | x | x |
| Watt et al.^(60)^ | Fair |  |  |  |  | x |  |  | x | x |
| Gregory et al.^(36)^ | Fair |  |  |  |  | x |  |  | x | x |
| Currie et al.^(61-63)^ | Poor-Fair |  |  | x |  |  |  | x | x | x |
| Sproston^(21)^  Craig et al.^(24)^  Craig & Hirani^(22)^  Craig & Mindell^(23)^ | Fair |  |  |  |  |  |  |  | x | x |
| Bromley et al.^(29)^ ^(26)^  Corbett et al. ^(27)^ ^(28)^ | Fair |  |  |  |  |  |  |  | x | x |
| Nelson et al. ^(55)^ |  |  |  |  |  | x |  |  | x | x |
| Euro-Urhis 2 ^(64-66)^ | Poor |  | x | x | x | x | x | x | x | x |

RQ = Research Question; Small sample size<100 participants (of either gender) in each region

| **Supplemental Table 6 – Quality Assessment – Adult Studies** | | | | | | | | | | |
| --- | --- | --- | --- | --- | --- | --- | --- | --- | --- | --- |
| **Author** | **Overall rating** | **RQ not clear** | **Population not clear** | **Response rate <50%** | **Recruitment not standardised** | **Sample size not justified/ small** | **Independent variable not valid** | **Dependent variable not valid** | **No cofounding adjustment** | **Limited statistical analysis** |
| Braddon et al.^(69)^ | Fair |  |  |  |  | x |  |  | x |  |
| Whichelow et al.^(76)^ | Fair |  |  |  |  |  |  |  | x | x |
| Gregory et al.^(35)^ | Fair-Good |  |  |  |  | x |  |  |  | x |
| MAFF^(52-54)^  Defra^(38-51)^ | Fair |  |  |  |  |  |  |  | x | x |
| Scarborough et al.^(9)^ | Fair- Good |  |  |  |  |  |  |  | Unclear |  |
| Haleem et al. ^(74)^ | Fair |  |  | x |  | x |  |  | x | x |
| Henderson et al. ^(31-33)^ | Fair |  |  |  |  | x |  |  | x | x |
| Ji et al.^(70)^ | Fair |  |  | x |  | x |  |  |  | Figures not presented |
| NatCen & UCL^(72)^ | Poor- Fair |  |  | x |  |  |  |  | x | x |
| NatCen & UCL^(71)^ | Fair |  |  |  |  |  |  |  | x | x |
| ScotCen^(68)^ | Poor- Fair |  |  | x |  | x |  |  | x | x |
| Sadler et al.^(73)^ | Poor-Fair |  |  | x |  |  |  |  | x | x |
| Nelson et al.^(55)^ | Fair |  |  |  |  | x |  |  | x | x |
| Shelton^(56)^ | Good- Fair |  |  |  |  |  |  |  |  |  |
| Craig et al.^(24)^  Craig & Hrani^(22)^ Craig & Mindell^(23)^ | Fair |  |  |  |  |  |  |  | x | x |
| Corbett et al. ^(27; 28)^ Bromley et al.^(26)^ | Fair |  |  |  |  |  |  |  | x | x |

RQ = Research Question; Small sample size<100 participants (of either gender) in each region
